# Allometry and Fighting Behaviour of a Dimorphic Stag Beetle *Cyclommatus mniszechi* (Coleoptera: Lucanidae)

**DOI:** 10.3390/insects11020081

**Published:** 2020-01-23

**Authors:** Zhen-Yi Chen, Yuying Hsu, Chung-Ping Lin

**Affiliations:** Department of Life Science, National Taiwan Normal University, No. 88, Sec. 4, Tingzhou Rd., Taipei 11677, Taiwan; 60543017s@ntnu.edu.tw (Z.-Y.C.); yuyinghs@ntnu.edu.tw (Y.H.)

**Keywords:** body size, male–male competition, mandible, sequential analysis, sexual selection, weapon

## Abstract

Male stag beetles (Coleoptera: Lucanidae) use their mandibles as weapons to compete for resources and reproduction. Mandible size in stag beetles can be associated with different behaviours and the outcome of male contests. We investigated the allometric relationship between mandible and body size in males of the stag beetle *Cyclommatus mniszechi* to uncover distinct morphs. The results divided male *C. mniszechi* into majors and minors with the switch point of mandible length at 14.01 mm. The allometric slope of mandibles was positive for both morphs but was steeper for the minors. We also characterised the fighting behaviour of the different morphs in size-matched contests using sequential analyses. Males matched each other’s behaviour in contests with many physical contacts, no injury and a progression from low towards high aggression and rare de-escalation. Major and minor males employed the same behavioural elements in contests, but major males were more likely to escalate directly into more aggressive phases and minor males tended to stay within phases. This finding suggests that major males may compete more aggressively than minor males in contests.

## 1. Introduction

Males of many insects use weapons to compete for resources essential for their survival and reproduction [1]. Sexually selected traits, such as ornaments and weapons, often evolve to reach extremes of size and elaboration [1,2,3,4]. One group of insects with an amazing diversity of weapons is stag beetles (Lucanidae). Male stag beetles use their exaggerated mandibles as weapons to fight for access to sap sites, territory and females [1,5,6,7]. Mandible and body size are reliable indicators of male stag beetles’ resource holding potential (RHP) and positively correlate with their ability to win in contests [6,7,8]. Stag beetles exhibit a high diversity in the sizes and shapes of mandibles both within and across species [1,9,10,11] which might cause the behaviour they display in fighting contests to be highly variable [1].

The scaling or allometric relationship between the sizes of different body parts and overall body size provides important information about an organism’s biology such as polyphenism [12], alternative reproductive tactics [13] and the nature of selection on different body parts [14,15]. The allometric scaling of sexually selected traits can be positively allometric (slope > 1), isometric (slope = 1) or negatively allometric (slope < 1) and often reflects resource-allocation trade-offs, the balance between natural and sexual selection and the genetic constraints on these traits [14,16]. Mandible and overall body size in many stag beetles show a linear positive allometry, in which larger males have relatively larger mandibles than smaller males [10,17,18,19,20]. Positive allometries suggest that larger males gain greater fitness advantages by investing disproportionally more in the development of weapons than other body parts [4]. Non-linear (curved, sigmoid or discontinuous) allometries between mandible and body size have also been reported for stag beetles (4,10,19). The declining slope of the curved allometry in the largest males is probably due to the depletion of resources available for the development of the weapon [15,19,21]. Sigmoid and discontinuous allometries are characterised with the evolution of size-dependent, alternative reproductive tactics [3]. The non-linear allometries between trait size and body size can be characterised following the approach and regression models recommended by Knell 2009 [22].

Understanding the nature of an animal’s fighting behaviour is essential to understand the evolution and diversification of its weapon forms [1]. How male stag beetles interact with and respond to each other in contests, however, remains poorly understood. For the majority of stag beetle species, it is unclear how males use their mandibular weapons in combat and whether males with different weapon sizes and shapes display different contest behaviours. There are approximately 1200 species of stag beetles in the world [23]. So far, fighting behaviour has only been reported for five species, namely, *Cyclommatus mniszechi* [8], *C. metallifer* [6], *Lucanus maculifemoratus* and *Prosopocoilus inclinatus* [24] and *Aegus chelifer* [7]. These studies indicate that the behavioural elements and sequences are highly variable both among and within species. The commonly used method for studying the contest interactions of stag beetles includes (1) placing two males some distance apart on a horizontal tree log [6,8] and (2) allowing the study animals a short time period (≤15–60 min) to acclimatise [6,7,8,24]. This method does not appear to be suitable for investigating the contest behaviour of *C. mniszechi* because 47% of the contest pairs failed to interact [8]. Different experimental methods, more closely resembling the study animal’s natural habitat, are therefore needed for studying the contest behaviours of different species of stag beetles.

**Figure 1:**
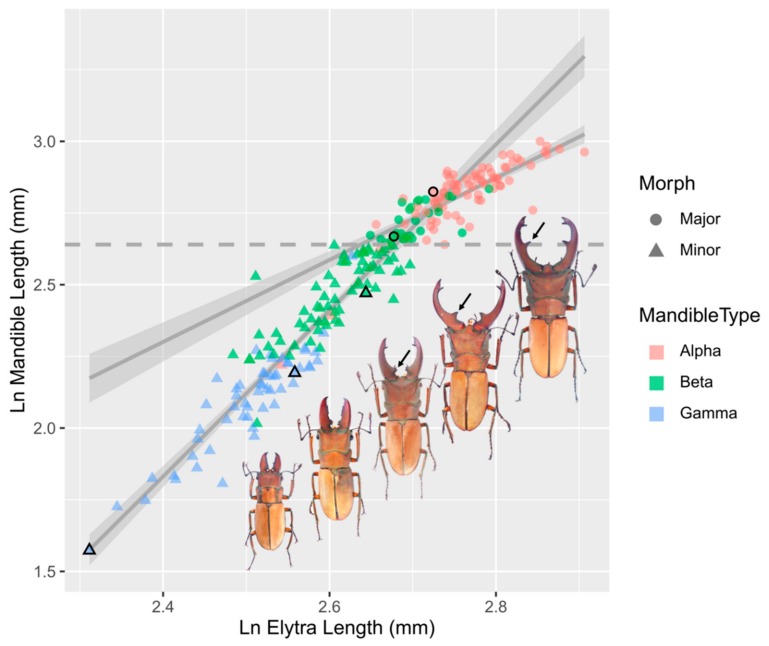
The allometric relationships between ln-transformed mandible length (ML) and elytra length (EL) of *Cyclommatus mniszechi* based on 34 collected and 198 reared males (*n* = 232). Solid grey lines are the linear regression lines of the major (circle, slope A = 1.43 ± 0.10) and minor (triangle, slope A = 2.89 ± 0.10). The 95% confidence intervals are shown in light grey. The dashed grey line is the switch point of ln mandible length at 2.64 (14.01 mm) based on Kotiaho and Tomkins [25] continuous piecewise model. In this study, males are divided allometrically into majors and minors above and below a mandible length of 14.01 mm, respectively. The black arrows point at the denticles on the mandibles which were used to classify males into alpha, beta and gamma morphs [8]. Alpha males have the largest mandibles with tusk-like projections (denticles) at their distal half. Beta males have smaller mandibles with denticles at their proximal halves and close to the base of the mandibles. Gamma males have the smallest saw-like mandibles with no apparent denticles. The major males contain mostly alpha morphs, and the minor males include beta and gamma morphs. The circles and triangles with black edges indicate the data points of the five beetles to their right.

This study investigated the allometry and fighting behaviour in the stag beetle *C. mniszechi*. An earlier study classified male *C. mniszechi* into three morphs based on the shape and size of their mandibles (Figure 1, alpha, beta and gamma) [8]. The relationship between mandible and overall body size of these beetles, however, remains unclear. We first examined the relationship between mandible and body size to identify which type of allometry best describes the relationship and determine whether the males could be grouped into different morphs based on the relationship. The allometric slopes of male morphs provide important information on the level of resource allocated to production of weapons. They are also useful to examine differences in resource allocation and fighting behaviours and the relationship between them in the different morphs. We then characterised the behaviour of males of different morphs in size-matched contests using sequential analyses. Size-matched contests allowed us to remove the effect of differences in males’ mandible size (a proxy for RHP) on contest outcomes and behaviours in order to characterise more precisely the behavioural difference among male morphs. This experimental approach has not been employed by previous studies of stag beetles’ fighting behaviours [6,7,8,24]. To study the contest behaviours of the males, our trials were novel in their experimental setups which allowed the males to encounter each other at the feeding site to compete for food, resembling the competition occurring in their natural habitat.

## 2. Materials and Methods

### 2.1. Study Organism

The stag beetle *C. mniszechi* is distributed in southeast China, Vietnam and northern Taiwan and inhabits lowland forests below approximately 750 m [26]. During the breeding season, from May to August in Taiwan, males perform mate-guarding behaviours and engage in territorial fights at the sap sites on the branches or trunks of broadleaf trees including *Fraxinus griffithii* (Oleaceae), *Broussonetia papyrifera* (Moraceae), *Citrus* spp. (Rutaceae) and *Koelreuteria elegans* (Sapindaceae). Male *C. mniszechi* are highly variable in body size, and an earlier study [8] classified them into three morphs (alpha, beta and gamma) based on the size and the shape of their mandibles. Alpha males have the largest mandibles with tusk-like projections (denticles) at their distal half (Figure 1, black arrows). Beta males have smaller mandibles with denticles at their proximal halves and close to the base of the mandibles. Gamma males have the smallest saw-like mandibles with no apparent denticles.

### 2.2. Insect Rearing

Adult *C. mniszechi* were collected from lowland forests of northern Taiwan between 2016 and 2018 with light traps and by chopping decayed wood and manually searching at sap sites on tree trunks. The 72 males collected from the field included 27 (37.5%) alpha, 34 (47.2%) beta and 11 (15.3%) gamma morphs. Males collected in 2018 were used in fighting contests the same year, and the males collected in 2016 and 2017 were used in breeding and morphological measurement. Field-collected females and males (after use in contests) were bred in the laboratory. Some reared larvae were obtained from local insect breeders in 2016 and 2017, and they were used for breeding and morphological measurement. Adult stag beetles were kept individually with an adequate supply of insect jelly (PPS-801, Champ E Pets Corporation, Taipei, Taiwan) in plastic containers (15 cm × 10 cm × 11 cm) at 25 °C under a 12 h:12 h (light:dark) cycle. The 2016 larvae were reared in 250 mL cylindrical plastic containers (7.5 cm in diameter; 4.5 cm in height) filled with fermented sawdust (good-quality microparticle fermented oak sawdust, Max Piggyfat Insect Feeding Facilities, Taiwan) under a 12 h:12 h (light:dark) cycle at 25 °C. Synchronous metamorphosis of beetle pupae can be regulated by chemical signals, social cues and the fluctuation of temperature [27,28]. The temperature (15.8–25.3 °C) used for larval rearing from September of 2017 to May of 2018 was therefore adjusted every two weeks (minimising the detrimental effect of sudden changes in temperature on the beetle’s health) to match the 2012–2016 mean monthly temperature at the Central Weather Bureau stations closest to the collection sites (Cyuchih (70 m) and Shanjia (48 m)) to synchronise the emergence time of adult males in 2018 for subsequent use in fighting contests.

### 2.3. Morphological Measurements

The mandible length (ML), head width (HW) and elytra length (EL) of the beetles were measured to the nearest of 0.01 mm using a digital calliper (99MAD027M1, Mitutoyo, Kanagawa, Japan). Mandible length was the average linear distance between the distal tip of the mandible and the axis of the mandibular joint of the two mandibles [7,8,29]; HW was the linear distance between the tips of the protrusions anterior to the eyes; EL was the linear distance between the posterior ends of the scutellum and the elytra. These three measurements were obtained on the day on which the beetles were collected in the field or the 21st day after the eclosion of the beetles reared in the laboratory. The body weight (BW) of the beetles was measured to the nearest of 0.001 g using a digital scale (CT-50, HIRODA, Shenzhen, China) one day before the contests.

### 2.4. Allometry Analyses

The morphological measurements of a total of 232 males were used for allometry analysis (Table S1). The plot of natural log of body size (elytra length, EL; X) against the natural log of weapon size (mandible length, ML; Y) indicated that the relationship between them was continuous and non-linear (Figure 1). We thus followed Knell 2009 [22] to characterise possible non-linear allometries. We first tested for linearity using the quadratic model [30,31]:

lnY = β_0_ + β_1_ lnX + β_2_ lnX^2^ + ε

where β_0_ is the intercept, β_1_ and β_2_ are the regression coefficients and ε is the error. A coefficient β_2_ which significantly differs from zero would suggest a non-linear relationship. We then tested for the following statistical models [25,31] using Akaike (AIC) and Bayesian information criterion (BIC) for model selection:

Linear model:

lnY = β_0_ + β_1_ lnX + ε

Eberhard and Gutierrez continuous piecewise:

lnY = β_0_ + β_1_ lnX + β_2_ (lnX − lnX_0_) D + ε

Eberhard and Gutierrez discontinuous piecewise:

lnY = β_0_ + β_1_ lnX + β_2_ (lnX − lnX_0_) D + β_3_ D + ε

Kotiaho and Tomkins linear:

lnX = β_0_ + β_1_ lnY + ε

Kotiaho and Tomkins quadratic:

lnX = β_0_ + β_1_ lnY + β_2_ lnY^2^ + ε

Kotiaho and Tomkins continuous piecewise:

lnX = β_0_ + β_1_ lnY + β_2_ (lnY − lnY_0_) D + ε

Kotiaho and Tomkins discontinuous piecewise:

lnX = β_0_ + β_1_ lnY + β_2_ (lnY − lnY_0_) D + β_3_ D + ε

For the Eberhard and Gutierrez continuous and discontinuous piecewise models, where lnX_0_ is the switch point, D = 0 if lnX < lnX_0_ (minor males) or D = 1 if lnX ≥ lnX_0_ (major males). The best estimate of the value of lnX_0_ was determined by the maximum adjusted *R*-squared values of the regression following the method of Sugiura et al. 2007 [32]. If β_2_ was significantly different from zero, the slope of allometry changed at lnX_0_. If β_3_ differed significantly from zero, the allometry was discontinuous at lnX_0_. For the Kotiaho and Tomkins models, lnY_0_ of weapon size (mandible length) is the switch point.

The isometry test using the equation lnY = A lnX + lna, a linearised form of Y = aX^A^ [17], was conducted to examine the allometric slope A between mandible size (ML)/head width (HW) and body size (EL) of different male morphs. The significance of the difference between A and 1 was evaluated by two-tailed *t*-test. An allometric slope of A > 1 (positive allometry) indicates that larger males have disproportionally larger mandibles than those of smaller males. When A = 1 (isometry), the mandible size among males is exactly proportional to their body sizes. A slope of A < 1 (negative allometry) shows that larger males have disproportionally smaller mandibles than those of smaller males. All allometric analyses were conducted in R (version 3.6.0, R Development Core Team, 2019) using the R scripts of Kojima and Lin 2017 [15].

### 2.5. Male–Male Contests

**Figure 2:**
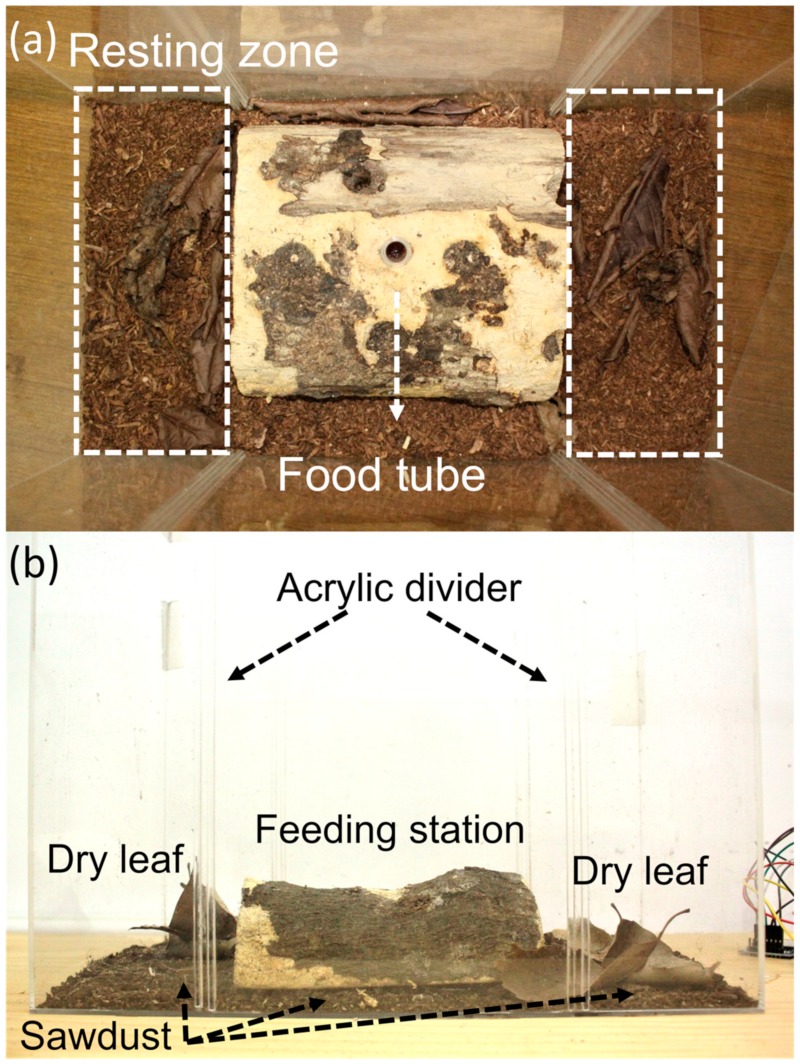
The fighting arena of *Cyclommatus mniszechi* used in this study: (**a**) overhead view, and (**b**) front view.

Size-matched contests were used to characterise the fighting behaviour of different males of *C. mniszechi* after eliminating the effect of unequal RHP. Because mandible size is used as a proxy for RHP in stag beetles [6,8], males reared in 2018 with similar mandible lengths were paired up for the contests. We only considered the fights staged between major and minor males because our analyses divided individuals of the beetles into these two groups (see results of allometry). We staged two types of contests (major versus major, minor versus minor) to investigate whether these two types of males use different fighting strategies. The difference in mandible length between the contestants was less than 5% (0.683 mm) of the median mandible length (13.655 mm) of all contestants (mean ± SD = 13.665 ± 3.126 mm, *n* = 114). The fighting arena was an acrylic container (32 cm × 18 cm × 30 cm) with 400 mL sawdust (1 cm height) at the bottom (Figure 2). The arena was divided into one fighting (16 cm × 18 cm) and two resting (8 cm × 18 cm) zones using two acrylic dividers (18 cm × 30 cm). Three to four dry oak leaves (*Quercus glauca*, Fagaceae) were placed on the surface of the sawdust layer of the resting zones for the beetles to hide under. A piece of half-cut wood (10 cm × 16 cm × 5 cm) was placed at the centre of the fighting zone to serve as a feeding station to facilitate competition between the two males. The arena was placed in a room at 25 °C with a 12 h:12 h (light:dark) cycle. None of the males used in the fighting contests had any prior fighting experience, and their bodies were all intact with no damaged or missing body parts.

All contests were conducted in the evening (from 18:00 to 19:00), because *C. mniszechi* is nocturnal [8]. At 18:00 the day before contest day, the two male contestants were placed in the two resting zones (one male per resting zone) of the fighting arena to acclimatise. During the daytime the male stag beetles usually hid under the dry leaves in the resting zones without obvious body movement. At 18:00 of the contest day, a 2 mL tube filled with insect jelly (PPS-801, Champ E Pets Corporation, Taipei, Taiwan) was inserted at the centre of the feeding station (Figure 2). Then, the two dividers were removed simultaneously to allow the two males to interact. The two males usually emerged from under the dry leaves, moved around the arena and walked towards the feeding station within 5 to 10 min of the dividers being removed. Agonistic interactions occurred when two males encountered each other, usually on the feeding station. This contest setup allowed the males to encounter each other during foraging/feeding and decide how to interact with each other. It resembled the situation that the males face in their natural habitat. The males were allowed to interact and fight until the fights were resolved with a clear winner and loser. If the two males did not exhibit sufficient aggression towards each other to produce a clear winner and loser, the trials were terminated after 1 h. After the termination of the trials, the two males were removed from the arena. The contests were recorded using night-vision video monitors (DS-VR7160H, Der Shuenn, Taipei, Taiwan) positioned 65 cm above the arena.

### 2.6. Sequential Analyses of Fighting Behaviour

We used BORIS v. 7.4 (Behavior Observation Research Interactive Software) [33] to transcribe the fighting behaviours of the males from the videos based on nine behavioural elements (Table 1). The contests were characterised as less (0: no “tussles”) or more intensive (1: with “tussles”). The fighting behaviours were coded individually in two adjacent columns, “preceding behaviour” followed by “subsequent behaviour”, for all behavioural transitions. The behavioural sequence data were summarised into adjacency matrices of contest behaviours using igraph network analysis package [34] in R following Green and Patek 2018 [35] (Tables S2 and S3). The transition rate for each behavioural transition was calculated from the ratio of the preceding behaviour to its subsequent behaviour. To examine whether a specific behavioural transition was more frequent than expected by chance, a permutation procedure was conducted by fixing the 1st columns (preceding behaviour), keeping the relative frequency of the behaviours and randomly sampling the 2nd columns (subsequent behaviour), thus randomising behavioural transitions between the behaviours [35,36]. The permutation process was executed 10,000 times to generate a null distribution of random transitions. A significant transition was found when the observed transition was more frequent than the respective 95% quantile of the null distribution of random transitions. Significant behavioural transitions of the contests were plotted as network graphs in igraph. The behavioural elements were organised into four phases (Table 1) based on the network graphs. We defined a phase as a behaviour or subset of behaviours, in which these behaviours had relatively similar level of aggressiveness and they were used with relatively equal frequency [35].

### 2.7. Statistical Analyses

The regression models were analysed in JMP (ver. 10.0.0, SAS institute Inc., Cary, NC, USA). A logistic regression model was used to examine the effect of either male morphs or mandible length on the level of aggression (probability of tussle) for all contests. Mandible length was used as the single explanatory factor in regression analyses of the contests of different male morphs.

## 3. Results

### 3.1. Allometry

For the allometric relationship between the mandible and elytra length, the β_2_ coefficient in the quadratic model was significantly different from 0, suggesting that the relationship was significantly non-linear (Table 2). The Kotiaho and Tomkins continuous and discontinuous piecewise model had the lowest AIC and BIC score, respectively. The Kotiaho and Tomkins discontinuous piecewise model improved the model fit only slightly (ΔAIC = 0.17), and the β_3_ coefficient of the model was not significantly different from 0, so the simpler Kotiaho and Tomkins continuous piecewise model was considered to be the best fit model for the allometric relationship. The value of the switch point (lnY_0_) which showed the highest adjusted R^2^ in this model was 2.64 (14.01 mm). The β_2_ coefficient of this model was significantly different from 0, indicating that the linear slope between the mandible and elytra length is continuous but changes markedly either side of the switch point (ML = 14.01 mm) (Figure 1). Therefore, male *C. mniszechi* are dimorphic and can be divided allometrically into majors and minors above and below a mandible length of 14.01 mm, respectively. The major males contain mostly alpha morphs, and the minor males include beta and gamma morphs (Figure 1). The allometric slope of the mandibles of minor males (A = 2.89 ± 0.10) is steeper than that of major males (A = 1.43 ± 0.10). The mandibles and heads of both major and minor males showed significantly positive allometries (Table 3).

### 3.2. Behavioural Sequences of Fighting Contests

A total of 55 size-matched contests (24 major morphs and 31 minor morphs) were staged. Males of 14 (25.5%) contests escalated to more aggressive “tussle” behaviour. Three (5.5%) contests were resolved without aggressive interactions (i.e., “push”, “attack”, “tussle” or “clamp”). The average duration of the size-matched contests was 37.2 ± 58.8 s (mean ± SD). No observable injury was found on any male contestant.

**Figure 3:**
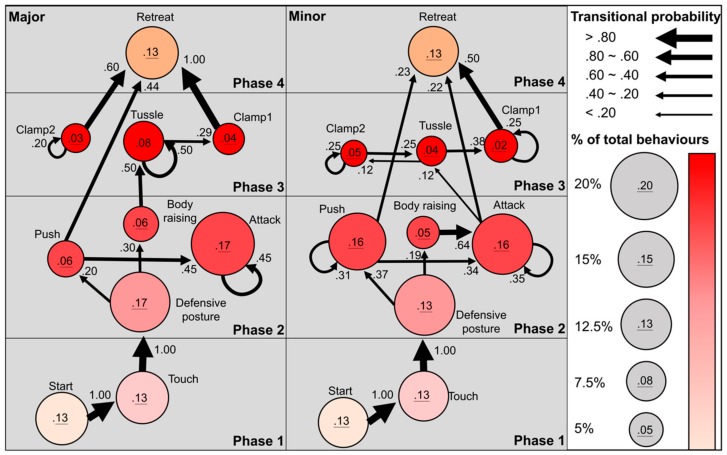
Sequential analyses of the contest behaviours of the major (*n* = 24) and minor (*n* = 31) males in size-matched contests of *Cyclommatus mniszechi*. Circles represent behavioural elements; circle size and colour are scaled to the percentage of total contest behaviours. Arrows represent significant behavioural transitions; non-significant behavioural transitions are not shown; arrow width is scaled to the transitional probability of the behaviours. Phases are coloured for visualisation.

The fighting contests of *C. mniszechi* stag beetle followed a variable course of behavioural sequences (Figure 3) (Video S1). The contest started when one male approached and touched its opponent’s body with its mandibles or forelegs (100%, transitional probability from “start” to “touch”, Figure 3). After “touch”, both major and minor males often entered a “defensive posture” by raising their heads and keeping their mandibles open towards their opponents (100%). “Defensive posture” then progressed to either “body raising” with rapid movement of the antenna (19%–30%) or “push” by raising mandibles and knocking the opponents (20%–37%). From “push”, the contests in both male morphs were either resolved by loser males entering “retreat” (23%–44%) or continuing into “attack” by biting the opponents with mandibles (34%–45%). From “body raising”, the behavioural sequences differed between major and minor males. In major males, “body raising” often directly led to “tussle” (50%) of phase 3 by interlocking mandibles and pushing each other, but it frequently resulted in “attack” of the same phase 2 in minor males (64%). Within phase 2, “push” was repeated many times in minor males (31%) but not in major males. “Attack” led to “tussle” (12%) and “retreat” (22%) in minor males, whereas “attack” was mainly repeated in major males (45%). In phase 3, minor males performed repetitive clamping onto the head (“clamp1”, 25%) and body of their opponents (“clamp2”, 25%) and frequent behavioural transitions between “tussle” and “clamp2” (12%–25%), while major males displayed repetitive “tussle” (50%) and “clamp2” (20%). From “tussle” of phase 3, the contests were often resolved by winner males clamping their rivals (“clamp1”, 29%–38%) and then flipping them into “retreat” (50%–100%).

Overall, the fighting behaviours of *C. mniszechi* progressed from phase 1, approach and touch; phase 2, defence and attack; phase 3, clamp and tussle and finally to phase 4, contest resolution (Figure 3). Behavioural matching between contestant males was observed for most behavioural elements. The contests of *C. mniszechi* males appeared to progress from less to more aggressive phases (e.g., from phase 1 to phase 2 or from phase 2 to phase 3) with a few behavioural transitions occurring within the same phases (Figure 3). Once contests escalated to aggressive physical contact (i.e., “push” and “attack” of phase 2; “clamp” and “tussle” of phase 3), behaviours frequently transitioned within phase or to phase 4 of contest resolution without first de-escalating to non-physical interactions in phase 2 (i.e., “body raising” or “defensive posture”). Minor males had more frequent behavioural transitions within phases than major males (i.e., between “body raising” and “attack”, and repetitive “push” of phase 2; between “tussle” and “clamp2”, and repetitive “clamp1” of phase 3, Figure 3). These results suggest that minor males tend to stay within these phases more than major males, whereas major males were more likely to escalate directly into more aggressive phases.

### 3.3. Male Morph and Contest Aggression

The regression analyses showed that the probability of tussle was not correlated with either mandible length (estimate ± SE = 0.185 ± 0.106, Chi-square = 3.02, *p* = 0.082) or male morphs (estimate ± SE = 0.344 ± 0.621, Chi-square = 0.31, *p* = 0.579) for all contests (*n* = 55). In the contests between minor males, the probability of tussle had a significant positive relation with mandible length (estimate ± SE = 0.709 ± 0.361, Chi-square = 3.86, *p* = 0.049, *n* = 31), whereas no significant relationship was found in the contests between major males (estimate ± SE = 0.341 ± 0.310, Chi-square = 1.21, *p* = 0.271, *n* = 24). These results indicate that neither mandible length nor male morphs have a significant effect on the probability of entering tussle. However, within minor morphs males with larger mandibles behaved more aggressively and were more likely to engage in tussles.

## 4. Discussion

Positive allometries were found for the mandibles and heads of *C. mniszechi* males. Allometric analyses demonstrate that *C. mniszechi* consists of dimorphic males defined quantitatively as majors and minors by mandible size at the switch point (Figure 1). The major males are mainly alpha morphs with the exception of a few beta morphs, whereas the minor males include both beta and gamma morphs, suggesting that the beta and gamma morphs have similar allometric slopes. The allometric slope of the mandibles of the major males is not as steep as that of the minor males, indicating that the major males invest proportionally less in the development of mandibles. A reduced allometric slope in the weapons of larger males is also found in other stag beetles [19], rhinoceros beetles [37,38] and flower beetles [15]. This pattern can be explained by resource exhaustion for pupal development of weapons [19,21], resource allocation between weapons and other body parts [39,40] or constraints of natural selection on costs in locomotion [41] and predation [42]. Alternatively, the difference in allometric slopes of mandibles between male morphs of *C. mniszechi* could be explained by the biomechanical functions of using mandibles as threat signals versus weapons [43]. The mandible opening behaviour of *C. mniszechi* could be used as a threat display to amplify the size difference between opponents. Whereas the functional advantages of a longer mandible may come from larger biting force or an enhanced ability to reach the rivals’ bodies to detach them. The reduced allometric slope in the mandibles of major males in *C. mniszechi* may represent a trade-off between these different functions [44].

We identified nine distinct behavioural elements in the fighting contests of *C. mniszechi* (Table 1), while an earlier study of the same species identified eleven distinct behaviours [8]. Eight of the eleven behaviours in Kuan 2011 [8] were observed in this study, the exceptions being “walk”, “approach” and “stand still”. The presence of these three behaviours may be an artefact of the trials being conducted directly on a horizontal tree log [8]; they were also observed in the fighting of *C. metallifer* stag beetles which used a similar setup [6]. We also observed a distinct “defensive posture” behaviour and separated “clamp” behaviour into “clamp 1” (head clamp) and “clamp 2” (thorax or abdomen clamp) to reflect the difference in the attacked body parts. “Encounter” in Kuan 2011 [8] was re-defined as ‘touch’ in our study to emphasise the physical contact between contestants. For *C. mniszechi*, “attack”, “push”, “clamp” and “tussle” are the four major fighting tactics used by males while competing with rivals. Among these actions, “attack” and “push” serve to keep the opponents away from the defended resources; “tussle” and “clamp” serve to flip the opponents or to dislodge them from the tree log. The same fighting tactics were also reported in congeneric *C. metallifer* stag beetles [6]. In addition, a peculiar shaking behaviour was observed in some winner males of *C. metallifer* immediately after the contests [6]. This shaking (pumping) behaviour was interpreted as intimidation by the winner male to display its dominance over the loser. Our observations in this study found that a few winner *C. mniszechi* males sometimes perform a similar shaking behaviour by lifting their wide-open mandibles up and down several times right after the contests. We suggest that this shaking behaviour in *C. mniszechi* may be an alert rather than intimidation behaviour because, immediately after the contests, the winner males were still very sensitive (motivated) and often responded to stimuli caused by the movement of loser males in the fighting area. The shaking behaviour might be involved in producing a substrate-borne vibrational signal which requires further study.

Our results suggest that the major and minor males of *C. mniszechi* employ the same behavioural elements in the fighting contests, but they differ in the extent to which they stay within phases in the contests and the likelihood of escalating into more aggressive tussle behaviour. Although the regression analyses found no significant effect of male morphs on the probability of tussle, the sequential analyses indicated that the major males were more likely to tussle and escalate directly into more aggressive phases, whereas the minor males tended to stay within the same phases. Our result contrasts with the observation in another stag beetle species, *A. chelifer*, in which the minor males tend to escalate more frequently than the major males [7]. Our findings suggest that the major males of *C. mniszechi* may have been selected to be more aggressive than minor males to compete in contests. The higher aggressiveness of major males in contests may be associated with using mandibles as weapons over threat signals to apply physical force and flip the rivals [43]. Within the minor morphs of *C. mniszechi*, the regression analysis indicated that males with larger mandibles were more likely to tussle which is consistent with the expectations from this weapon hypothesis. The elevated aggression in the major males can also be explained by their higher investment in the production of relatively larger weapons. The loss of fitness benefit through withdrawing from contests may have a higher cost for the major males, so they tend to be more aggressive than the minor males in contests. Larger males of stag beetles with longer weapons (more muscle mass) have disproportionally higher resting metabolic rates than smaller males with shorter weapons [45].

## 5. Conclusions

Stag beetles are charismatic insects known for displaying extremely diverse sizes and shapes in mandibles used as weapons in male contests for resources and reproduction. Size variation of stag beetles’ mandibles is important for determining their fighting behaviours and the outcomes of contests. Here, we found that two distinct male morphs of *C. mniszechi* employ the same behavioural elements but differ in their behavioural sequences in size-matched contests. Major males with larger mandibles are more likely to tussle and escalate directly into more aggressive phases, whereas minor males with smaller mandibles tend to stay within the same aggressive phases. Further research is needed to understand the underlying ecological and evolutionary mechanisms for producing this variation of contest behaviours.

## Figures and Tables

**Table 1:** Behavioural elements in the contest interactions among males of *Cyclommatus mniszechi*.

Behavioural Elements	Description	Phase
Touch	One of the contestants touches its opponent’s body with mandibles, antenna or legs.	1
Defensive posture	One of the contestants keeps its mandibles open and raises its head.	2
Body raising	Two contestants face each other and raise their bodies, thoraxes and mandibles with rapid movement of antennas and forelegs and move towards each other.	2
Attack	One of the contestants uses its mandibles to bite its opponent.	2
Push	One of the contestants raises its mandibles and knocks its opponent.	2
Tussle	Two contestants interlock each other’s mandibles and push each other.	3
Clamp 1	One of the contestants uses its mandibles to clamp onto the head of its opponent.	3
Clamp 2	One of the contestants uses its mandibles to clamp onto the body (thorax or abdomen) of its opponent.	3
Retreat	One of the contestants moves backwards and away from the other male.	4

**Table 2:** Model selection of the allometry between mandible length (ML) and elytra length (EL) in *Cyclommatus mniszechi*.

Parameter	Estimate	SE	*p*	AIC	ΔAIC	BIC	ΔBIC
Linear model							
β_0_	−4.333	0.131	<0.001	−471.06	503.32	−460.73	499.69
β_1_	2.591	0.049	<0.001				
Quadratic model							
β_0_	−20.250	2.297	<0.001	−513	461.38	−499.56	460.86
β_1_	14.698	1.745	<0.001				
β_2_	−2.298	0.331	<0.001				
Eberhard and Gutierrez continuous piecewise							
β_0_	−5.372	0.165	<0.001	−535.14	439.24	−521.35	439.07
β_1_	2.997	0.063	<0.001				
β_2_	−1.627	0.187	<0.001				
Eberhard and Gutierrez discontinuous piecewise							
β_0_	−5.367	0.188	<0.001	−533.14	441.24	−515.91	444.51
β_1_	2.994	0.073	<0.001				
β_2_	−1.633	−1.633	<0.001				
β_3_	0.001	0.019	0.95				
Kotiaho and Tomkins linear							
β_0_	1.747	0.017	<0.001	−931.40	42.98	−921.06	39.36
β_1_	0.356	0.007	<0.001				
Kotiaho and Tomkins quadratic							
β_0_	2.359	0.114	<0.001	−957.69	16.69	−943.91	16.51
β_1_	−0.156	0.094	0.0987				
β_2_	0.105	0.019	<0.001				
Kotiaho and Tomkins continuous piecewise							
β_0_	1.860	0.023	<0.001	−974.21	0.17	−960.42	0
β_1_	0.305	0.010	<0.001				
β_2_	0.202	0.029	<0.001				
Kotiaho and Tomkins discontinuous piecewise							
β_0_	1.877	0.025	<0.001	−974.38	0	−957.15	3.27
β_1_	0.297	0.011	<0.001				
β_2_	0.179	0.033	<0.001				
β_3_	0.011	0.007	0.144				

**Table 3:** Parameters estimated for the isometry tests in *Cyclommatus mniszechi* (ML = mandible length, HW = head width, A = allometric slope, lna = intercept).

Morph	Traits	Coefficients	Estimate ± SE	95% Lower Limit	95% Upper Limit	*p*
Major	ML	A	1.43 ± 0.10	1.24	1.62	<0.001 *
		lna	−1.13 ± 0.27	−1.66	−0.60	
	HW	A	1.30 ± 0.07	1.17	1.44	0.001 *
		lna	−0.94 ± 0.18	−1.30	−0.57	
Minor	ML	A	2.89 ± 0.10	2.69	3.10	<0.001 *
		lna	−5.12 ± 0.27	−5.65	−4.58	
	HW	A	1.97 ± 0.06	1.86	2.08	0.001 *
		lna	−2.77 ± 0.14	−3.06	−2.49	

* *p*-value < 0.05.

## Supplementary Materials

The following are available online at https://www.mdpi.com/2075-4450/11/2/81/s1, (Version 2.0, 10.5281/zenodo.3603560): Table S1: The morphological measurements of males of *Cyclommatus mniszechi* used for allometry analyses. Table S2: The behavioural sequence data used for sequential analyses of size-matched contests in major males of *Cyclommatus mniszechi*. Table S3: The behavioural sequence data used for sequential analyses of size-matched contests in minor males of *Cyclommatus mniszechi*. Video S1: The behavioural sequence of males of *Cyclommatus mniszechi* in fighting contests under the laboratory setup. One of the opponents walked to the other and touched it (00:07), and then both of them displayed “defensive posture” (00:08) after “touch”. Once both individuals approached each other, they accelerated antennation and raised their mandibles and prothoracic parts. The contest then progressed into “body raising” (00:14). They then performed “attack” and “push” on each other several times and then escalated to “tussle” (00:20) and interlocked their mandibles until one of the contestants was clamped (“clamp1”) in the air by the other for a second and flipped (00:50). The winner dropped the loser and kept attacking and pushing the loser, while the loser retreated and moved backwards (00:51).
